# How a (sub)Cellular Coincidence Detection Mechanism Featuring Layer-5 Pyramidal Cells May Help Produce Various Visual Phenomena

**DOI:** 10.3389/fpsyg.2015.01947

**Published:** 2015-12-22

**Authors:** Talis Bachmann

**Affiliations:** University of TartuTartu, Estonia

**Keywords:** consciousness, neural correlates of consciousness, visual perception, illusions, phenomenology, neuromodulation, dendrites, pyramidal cells

## Abstract

Perceptual phenomena such as spatio-temporal illusions and masking are typically explained by psychological (cognitive) processing theories or large-scale neural theories involving inter-areal connectivity and neural circuits comprising of hundreds or more interconnected single cells. Subcellular mechanisms are hardly used for such purpose. Here, a mechanistic theoretical view is presented on how a subcellular brain mechanism of integration of presynaptic signals that arrive at different compartments of layer-5 pyramidal neurons could explain a couple of spatiotemporal visual-phenomenal effects unfolding along very brief time intervals within the range of the sub-second temporal scale.

## Introduction

Non-veridical subjective experiences of external physical reality are typical for situations where very brief visual stimuli interact within a sub-second time interval. Spatial, temporal and spatio-temporal distortions and misrepresentations are usually the essence of these phenomena (Bachmann et al., 2011). Characteristically, when pairs or multiples of brief stimuli in the range of dozens of milliseconds (ms) are presented with comparably short intervals, subjective delays with which target stimuli appear in explicit perception often tend to misrepresent the objective temporal relations between targets and reference stimuli. Moreover, when masking is the case the stimuli that are phenomenally distinct and clear when presented alone become deprived of conscious experience when paired with perceptual masks. In this paper I will describe a couple of such phenomena suggesting a common subcellular level neural mechanism hypothetically capable of explaining these phenomena. The core of the mechanism consists in a coincidence detection operation performed by certain subcellular and synaptic processes. It is my belief that this kind of approach in principle is consistent with the emerging *Zeitgeist* of nanophysiology which is set to deal with processes taking place between different compartments of single cells (Holcman and Yuste, 2015).

## The Generic Mechanism of Coincidence Detection

Most of the theoretical approaches adopted for explaining the temporal illusions and masking have been based on cognitive mechanisms, systems level neural architectures or neural population level models. However, in several theoretical models of explicit perception the core of the model is implemented at the cellular and sub-cellular levels of sensory processing. The temporal parameters and functional underpinnings of the models allow use them to explain several common temporal illusions. These models have helped to explain binding of features for formation of integrated perceptual objects and also to explain how pre-consciously processed sensory information becomes bound with contextual representations, thus becoming incorporated into the consciously experienced scenes. Specifically, the list includes the LAMINART model by Stephen Grossberg (e.g., Grossberg and Versace, 2008), the model developed by Rodolfo Llinás and his colleagues (e.g., Llinás et al., 1998), the perceptual retouch model by Bachmann (1994, 1997), the zero-lag synchronization mechanism by Raul Vicente and colleagues (e.g., Vicente et al., 2008), and the backpropagation-activated Ca^2+^ spike firing or *BAC firing* mechanism by Matthew Larkum and associates (e.g., Larkum et al., 1999; Larkum, 2013). The characteristics of the listed mechanistic models essential for the view advocated in this paper are:

(i) perceptual contents are encoded by the neocortical pyramidal neurons (PN);
(ii) afferent presynaptic input from stimulation becomes explicitly perceived when the activity of thalamo-cortical microcircuits that represents specific sensory contents is synchronized with the activity of thalamo-cortical non-specific modulatory microcircuits; this system involves layer-4 PN that are activated mostly by first-order and higher-order feedforward connections from thalamus and layer-5 PN as an essential link in integrating the cascaded thalamo-cortico-thalamic processes by sending cortico-thalamic efferents to higher-order thalamic relays such as pulvinar;
(iii) modulatory presynaptic input essential for long-range integration (top–down connectivity) and contextual effects from higher level cortex and/or from non-specific reticulo-thalamic alerting system arrives at the apical dendrite compartment of PN;
(iv) for perceptual integration to be available for conscious experience (representing objects and scenes), temporal coincidence of the somatic presynaptic input and apical-dendritic presynaptic input is necessary;
(v) the varying contents of explicit perception are extracted and selected from the active type-PN neurons by the coincidence detection mechanism; this mechanism makes part of the available information accessible for consciousness;
(vi) this cellular/subcellular mechanism is subject to neuronal plasticity effects in learning and constitutes a device where actual sensory input and contextual information from long- and short-term memory interact;
(vii) the temporal dynamics of activity of the PN units is explicated in excitatory post-synaptic potentials (EPSPs) and spiking as dependent on the level of EPSP depolarization; it is subject to varied inhibitory effects by GABA-ergic interneurons;
(viii) feed-forward sensory effects are somatic-compartment driven whereas top–down and collateral modulation effects are apical dendrite (including tuft) compartment driven.

Information contents of objects and scenes are encoded in the multiple modules of cortex, ordered along the afferent processing stages retina-LGN-V1-V2-V4-IT. However, most of information reaching these cortical areas benefits from the cascaded system of thalamic relays (Sherman, 2005). It is widely accepted that layer-4 and -3 pyramids are the main stuff of cells that are tuned to the specific contents of stimulation represented in V1, V2, V3, V4, V5/IT (Guillery and Sherman, 2002; Sincich and Horton, 2005; Sherman, 2007). While there is no evidence that layer-5 P cells directly respond to sensory input, these cells are crucial in allowing multi-featured stimulation responded to by layer-4 and -3 cells to be integrated for conscious perception where different features of objects are perceptually bound together (Llinás et al., 1998; Jones, 2001; Ribary, 2005). Moreover, there are several reasons that allow me to postulate that in addition to acting as an integrating device and modulator (including top–down effects from cortex to subcortex), layer-5 PN is a neural unit, activity of which may be a reliable signature of the specific contents of sensory processing reaching conscious-level representation. First, although primary geniculo-cortical afferents most massively target layer-4 PN, there are also specific sensory fibers to layer-5b pyramids (including the “tall” variety with long apical dendrites) (Callaway, 2004). Second, layer-5 PN axons target most of other layers except layer-4 (Callaway, 2004). However, because temporal delays between sensory input to a layer-5 PN and its driving effect on supragranular cells that receive input from layer-4 cells are extremely short it can be said that layer-5 activity virtually synchronously mimics content-specific layer-4 and supragranul activity. Third, if we accept the sparse coding principles in neocortical representation then it is well conceivable that despite its relatively less expressed specific afferent innervation compared to layer-4 and different supragranular cells, layer-5 neurons may well be capable of encoding the afferent contents of stimulation. Fourth, there is substantial fast-acting input from layer-5 PN to layer-4 higher order subcortical relays (Sherman, 2005) and thus a lower level layer-5 pyramid can signal a higher-order layer-4 PN activity. Fifth, because the calcium spike based BAC firing mechanism is extremely “explosive”, the relative small number of specific afferent presynaptic fibers targeting layer-5 PN can be compensated for by a rigorous burst of spiking (Larkum, 2013). Sixth, because layer-5 PN possess many widely branching axons, this type of cell can multiply relatively few early level presynaptic sensory signaling through diverging directions to higher levels of complex cells representing complex attributes of stimuli. Seventh, it is well known that early electrophysiological markers of neural activity correlating with conscious perception of specific contents of stimulation have a latency of about 100–150 ms or 200 ms (Bachmann, 1994; Railo et al., 2011; Schoenfeld et al., 2011; Navajas et al., 2013; Pitts et al., 2014; Andersen et al., 2015; Rutiku et al., 2015). This means that neural correlates of conscious-level perceptual activity attributed to layer-5 PN have to have a corresponding post-stimulus latency. Hansen et al. (2012) showed that both infragranular and supragranuar neural activity in V1 boosted after 100 ms whereas granular activity had a boost before 100 ms. As layer-5 specifies infragranular units, the association of surface-negative electrophysiological markers of becoming aware of stimulus contents suggests that apical amplification (which is characteristic to BAC firing mechanism of layer-5 neurons) helps bringing respective contents to consciousness.

Although layer-5 PN do not take any *direct* part in the feed-forward retina-LGN-V1 object representation and perception stream, the activity of layer-5 cells can be considered as a “litmus test” of whether certain contents carried by PN of other layers have been integrated to a conscious percept.

**FIGURE 1:**
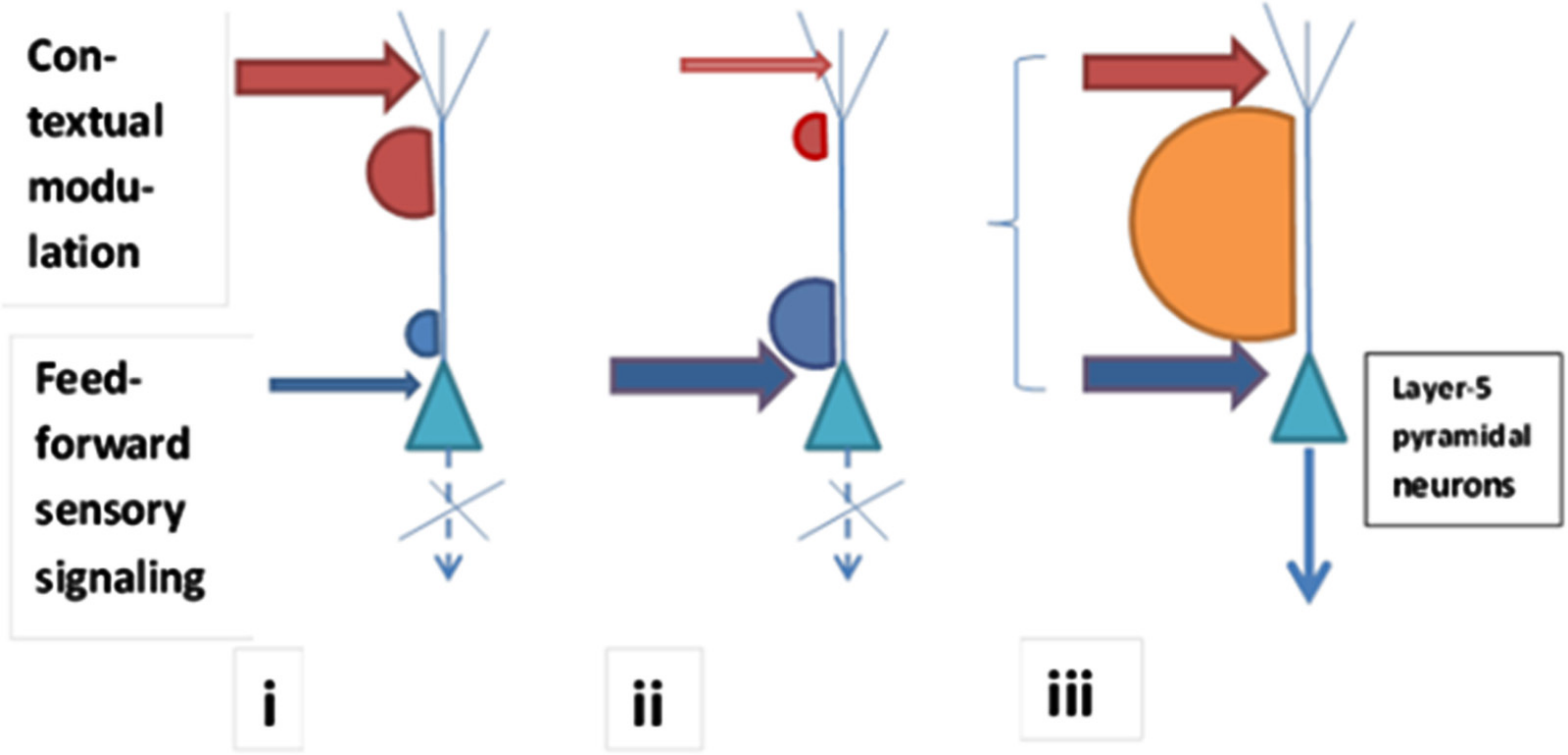
**Conscious access to the contents signaled by a layer-5 PN depends on whether somatic feedforward presynaptic input (signaling sensory contents of stimulation) is coincident with apical-dendritic presynaptic input (carrying contextual modulatory signals). (iii)** Two arrows symbolizing the arrival of feedforward (blue arrow) and modulatory (red arrow) input are aligned in time, which refers to temporal coincidence of somatic and apical inputs as a prerequisite of burst firing necessary for communicating the contents between levels. In this case BAC firing mechanism is ignited with its characteristic plateau wave allowing rigorous spiking **(iii)**. When somatic input is relatively strong (**ii**, blue arrow), but not accompanied by sufficient synchronous apical input (**ii**, red arrow), conscious access is absent [the pyramid in **(ii)** does not fire sufficiently]. When apical modulatory input is sufficiently expressed, but somatic synchronous sensory input is weak, conscious access to this sensory data is not possible (**i**). When in a non-conscious contents condition specific input is relatively substantial (**ii**), this content can be upgraded to conscious access fast because only a few synchronizing modulatory presynaptic apical inputs are needed. When in a non-conscious contents condition modulatory input is relatively substantial, but specific input weak (**i**) conscious access may be granted instead for a different PN that gets similarly strong apical modulation, but gets also a strong somatic input of a different content (e.g., **iii**, interpreted as referring to a different neuron from the one shown as **i**).

For the layer-5b PN to generate plateau-wave-based spiking, the temporal coincidence of somatic sodium channel-related presynaptic input and calcium channel-related presynaptic input targeted at the apical compartment of the cell is necessary (Larkum, 2013). The somatic input informs appearance or presence of specific sensory input and input to apical dendrite mediates modulation by the associative system. Importantly, suprathreshold input to the neuron’s body (responsible for signaling about the new stimuli) produces fewer action potentials of the cell than triggering of the dendritic Ca^2+^ spikes does. This substantiates the importance of modulatory brain processes in addition to the straightforward sensory afference and provides a convincing argument for the common effects of biased perception being under the contextual and arousal systems control. (See also Llinás et al., 1998, on the putative significance of coincidence detection for the effectively working consciousness mechanism.) **Figure 1** illustrates the key elements of this conceptualization.

I posit that *several temporal perceptual phenomena are due to the preconditions requiring that* (a) target stimuli are presented together with contextual stimuli, (b) temporal parameters of the duration and succession of the stimuli are compatible with the time constants of activity of the timing of presynaptic inputs and plateau waves of EPSPs of the coincidence detection mechanism, (c) the somatic presynaptic input of the layer-5 PN cells signaling about certain perceptual content and the dendritic apical compartment input of the *same* layer-5 cells can originate from *different* stimuli inputs, (d) the temporal delay to conscious access of the perceptual contents is determined by the slowest link in the hierarchical modules which represent the object and scene contents. This set of premises makes it possible that target stimulus’ contents-signaling activity of the PN is modulated by the contextual stimulation so that illusory shifts of perceptual delays of different content in real time may occur. Moreover, in some stimulation conditions these premises allow a stimulus that follows to substitute a preceding stimulus in conscious experience.

## The Phenomena to be Explained

### Temporal Reversal of Stimuli in Consciousness

Recently, a novel temporal illusion was presented by Wu et al. (2009) demonstrating that the cause of a perceptual event can be perceived after the event itself. In a motion-induced blindness (MIB) display, a static visual target presented on a constantly rotating background of small crosses disappears and reappears from awareness periodically. When a flash was presented during a period of perceptual suppression it almost instantaneously caused subjective reappearance of the suppressed target. However, although being the cause of the target’s reappearance (the subjective effect), the flash was systematically perceived as occurring after this reappearance. The subjective illusory delay to consciousness was about 100 ms. The authors did not present any specific coherent theory for explaining the effect and suggested that specific information updating and/or reentrant processing of subliminal target trace might be involved. Obviously, it must be a *non-specific* mechanism which brings the non-conscious specific representation to consciousness because any explanation requires a process that is activated by the flash but acts on the representation of the target (and is, therefore, not specific). In the coincidence detection based binding mechanism apical-dendrite presynaptic input implements the non-specific counterpart and somatic presynaptic input implements the specific target-content counterpart. As the target stimulus is physically present also when *subjectively suppressed* during MIB, by definition the apical presynaptic input to the PNt neuron representing the target should be insufficient during MIB although the somatic input for PNt must be sustained (presumably at the near-spiking threshold level of its EPSP, **Figure 1**, ii). When a flash is presented, its transient response evokes non-specific presynaptic input to apical dendrites of the PNt neuron and as the PNt related EPSP was already at the near threshold level, plateau-wave spiking of the PNt is ignited at once (**Figure 1**, iii). This leads to the conscious perception of the target. The sustained part of PNf signaling necessary for the flash-stimulus perceptual content representation takes time and only after a delay of about 100 ms specific somatic and contextual dendritic pre-synaptic inputs coincidence is processed, resulting in the delayed awareness of the flash. When the flashed object is presented, two processes are triggered – the process for representation of the contents of the flashed stimulus and the boost of or perturbation in the contextual process presynaptically alerting the PN neurons at their apical-dendrite compartment. This facilitated (or reset) contextual activity leads to binding of the already present pre-conscious PNt-activity with global consciousness-level representation. This binding process takes little time because there is no need for build-up of the content-specific neural representation for the target as it is pre-consciously already present. (In some computational model, only phase resetting between the already functioning two oscillatory activities is required.)

### Illusory Temporal Dissociation of Filled-in Shape Surface Quality and Background Sensory Quality

Another intriguing temporal illusion was presented by Motoyoshi (2007). An illusory object (with its edges formed by illusory contours) was briefly flashed on color background which gradually changed its hue. An illusory dissociation between the color of the object formed by illusory contours and its surrounding background color was perceived. The illusory object’s surface appeared subjectively as having the color of the background from the earlier time when the inducing elements of the illusory-contour object were flashed but the background surrounding the object appeared in the color the background obtained later in time. Again, the author did not present any specific coherent theory for explaining the effect and suggested a possible involvement of specific cortical filling-in mechanisms for static objects (working slower than the dynamic background signaling mechanisms). From the present theoretical point of view this illusion can be explained by the temporal coincidence of the two processing states. One is the slower object form related presynaptic somatic activity of the neurons PNcf at a higher level where color and form of the filled-in contoured object had been integrated. The other is the fast background color representing contextual activity driven by the contourless background color where somatic and apical compartment presynaptic coincidence has been processed faster. For the PNcf representing color/form integrated objects access to consciousness is slower and with regard to it the internal state iii (**Figure 1**) represents an earlier external time moment than the internal state iii (**Figure 1**) for the PNbg representing diffuse background color. This explanation either suggests that sensory consciousness emerges right there where the corresponding perceptual contents coding PN are located (even though at different cortical levels) or assumes a higher level integrative locus where “all comes together” with a temporal delay.

### Backwrad Masking

In backward masking, if a brief target stimulus (e.g., with 40 ms duration or less) is rapidly followed by a masking stimulus, target may not be consciously perceived and the masking stimulus is explicitly experienced instead (Bachmann, 1994; Breitmeyer and Öǧmen, 2006). Majority of masking theories explain this by some inhibitory mechanism where masking-stimulus signals suppress or interfere with target-stimulus signals (review: Breitmeyer and Öǧmen, 2006). The hypothetical picture of the BAC firing mechanism processes that would lead to this kind of behavioral/subjective outcome is as follows. First of all, let us remember that the theoretically necessary condition for sensory input to be integrated up to the level of conscious experience requires temporal coincidence of the input to different compartments of the same neuron. Input from feedforward sensory channels targeted at the somatic compartment of the PN cell that represents the signaled content and associative (modulating) input targeted at the distal dendritic compartment of the cell must simultaneously coincide in time. Target signals feed synaptic receiving membrane compartment close to cell soma with a short delay (say, 30–50 ms) generating few somatic Na^+^ spikes. However, because this delay is too short for any associative input to arrive to the tuft region of the dendrite in response to the target-evoked perturbation, initially there is no target experience. Processing is pre-conscious. After some more time has lapsed, this associative, tuft-area directed presynaptic input arrives (say, with about 100 ms post-target delay), but it coincides with the mask-stimulus evoked Na^+^ spikes produced by the neurons that encode mask features. Because certain features of target and mask are shared by the target-responsive and mask-responsive cells (e.g., spatial location, some blob-, or edge-defining features, etc), mask-responsive cells receive also the associative presynaptic Ca^2+^activity initiating input to the dendrite’s upper compartment (this was evoked by the preceding target). Because this input is coincident with Na^+^ spikes, a plateau wave is produced primarily for the neurons representing the mask features instead of the neurons representing exclusively the target features (**Figure 1**, iii). We must remember here that the associative input in response to a perturbation by a stimulus has a longer delay to reach the apical compartments of the dendrites of the neurons compared to the delay it takes for the initial basal input to arrive to the cell. This basic fact is the crucial precondition for BAC firing based binding of the later presented stimulus with conscious representation instead of the first presented, briefly offset stimulus. Thus, the target-evoked dendritic Ca^2+^ mediated EPSP appears after a delay, is spread also to dendrites of other cells (e.g., mask-related neurons), and coincides with the fast Na ^+^ based somatic EPSP/spiking process of the mask-related cells. It is exactly then and there when and where the coincidence detection device sets in, but as a result, the masking stimulus is emphasized for awareness. An open question here is this: what are the relative roles of the (i) specific top–down associative backpropagation from the higher nodes of representations of specific input and (ii) non-specific (“diffuse”) thalamocortical modulation mediated by the directed arousal system? Bachmann (1994, 1997) in his model of modulated EPSPs as the explanatory mechanism of masking emphasizes the latter aspect. Larkum (2013), in his explanation of feature binding, leaves open the role of non-specific thalamocortical input possibly targeting the same dendritic area (layer 1) as reentrant cortical feedback inputs do.

### Illusory Misbinding of Features

In certain specific mutual masking experimental protocols, illusory misbinding of features of the two successive, spatially overlapping targets occurs when intermediate stimulus onset asycnhronies (SOAs) separate targets in time. For example, this happens when S1 and S2 are stimulus-objects with two integrated within-object, task-relevant features in each (Hommuk and Bachmann, 2009). When certain shape (e.g., square, ring, triangle) is combined with certain orientation of the grating filling the area of that shape (e.g., vertical, horizontal, oblique) and when subjects are asked to report the contents of the object not by the target feature (according to which he/she searches that object), but according to the other feature associated with the target feature, illusory misbindings are typical. The shape of S1 (when target is searched according to that shape) is misbound with the surface-grating of S2, but rarely the opposite version of misbinding happens. According to the hypothetical coincidence detection (cellular level) mechanism the neural events leading to misbinding are as follows. Shape processing as a relatively higher level operation takes longer than grating orientation processing. The top–down activity directed from the PN neurons encoding S1 shape and targeted at the apical compartment of the dendrites of the lower level orientation encoding neurons produces Ca^2+^ membrane activity exactly at the time when the feedforward somatic input to the lower level PN neurons encoding S2 surface orientation arrives there. This temporal coincidence allows to produce a plateau-wave in the neurons that carry information about S2 surface orientation, which leads to perceptual integration of this feature with the higher level neural activity representing S1 shape. The result is illusory binding of S1 shape and S2 surface orientation in the perceived object. The temporal asymmetry of misbinding between the two attributes separated in time speaks against the response bias explanation of the illusion.

### Flash-Lag Efect

I will end the examples of illusions with the much celebrated flash-lag effect (FLE; Sheth et al., 2000; Nijhawan and Khurana, 2010; Bachmann, 2013; Hubbard, 2013). When an object continuously changes its feature value (e.g., location when in motion or color when presented from the same location) and an invariant object is briefly flashed, with its value matching the value of the changing object, the flashed object appears to lag behind the continuously changing object. For example, the moving object appears to be ahead of the flashed object along the trajectory of motion (even though actually the objects were aligned) or the color of the flashed object appears to have the hue the continuously color-changing object had a brief moment ago. Among the most popular theories explaining the FLE we should first of all refer to the extrapolation theory: the moving target is extrapolated in space and the flashed static reference is therefore lagging behind (see Nijhawan and Khurana, 2010). Another well known theory suggests that the moving stimulus is processed faster than the stationary flash, which also causes the flash to lag. However, FLE can be obtained also when a target is presented in a stream of spatially overlapping stimuli (target compared to an out-of-stream reference stimulus) (Bachmann and Põder, 2001). This invalidetes the theory based on differential processing speeds of moving and stationary stimuli. On the other hand, if the mechanism of exptrapolation is controlled by presenting an additional moving stimulus in order to cancel the motion vector of the moving reference stimulus, FLE is alive and well despite this nullification of the extrapolation activity (Bachmann et al., 2012). This result puts extrapolation theory as the only valid theory in doubt. From the point of view of the coincidence detection mechanism the effect appears as a result of the difference in the delays with which the crucial presynaptic input arrives at the PN neurons. Let us compare the delays with which the apical dendrite-targeted, presynaptic contextual input arrives at its presynaptic membrane locus compared to the time when the somatic presynaptic feedforward input arrives at its presynaptic membrane locus. In case of the continuously changing object the preceding instances of the sensory input have already succeeded in driving the time-consuming activity of the higher level nodes above PN level. Consequently, the reentrant contextual input targeted at the apical dendrites of the PN coincides in time with the newly arriving somatic presynaptic input to this cell. Because of this “stealing of time” the very first afferents of the continuously changing stimulus take part in coincidence based boosting of the plateau wave of the PN (e.g., a state as in **Figure 1**, i changes very fast to a state as in **Figure 1**, iii). The changing object’s perceptual values are fast to conscious perception. With the flashing object there is a temporal handicap: because the flashed object is a newly appearing one, it takes time to prepare the bottom–up plus top–down contextual reentrance for apical presynaptic input. This means that the somatic plus apical input coincidence detection for the PN of the flashed object has a longer delay. The same subjective moment in time presents the observer with different perceptual values of the changing and invariant objects: for the flashed feature this value comes from a bit more distant past while for the changing object it is more contemporary.

The present theoretical account capitalizing on the contextual modulation by the BAC firing mechanism has an advantage over the above mentioned alternative theories put forward for explaining the listed phenomena. Each of these theories is narrowly bound to certain one specific phenomenon while the present theoretical approach can explain a multitude of phenomena in a single theoretical framework. Sure enough, although the temporal parameters of the listed phenomena have a largely coinciding order of magnitude of the temporal scale (e.g., 50–200 ms), the precise timing values may differ. Therefore, in future research it is important to find the ways to test specific predictions related to precise timing of the phenomena vis-à-vis the timing of the subprocesses of the BAC firing, contextual modulation, mechanism. Although this is technically complicated with human subjects, one way would be to measure directly the BAC firing processes in the conditions across different experimental settings that produce these different phenomena. (If animal models of perceptual behavior – supposedly based on the analogous phenomena – could be developed, direct measurements by patch clamp or optogenetic procedures may be envisaged. First attempts along this direction can be noted: e.g., Manita et al., 2015). If the time constants of the BAC firing related activity of the neuronal motifs will change according to the stimulation context specific to each phenomenon, the generality of the present theory will receive support. On the other hand, it should be possible to modulate the timing characteristics of the BAC firing mechanism by psychopharmacological intervention targeted at modulating the neurotransmitter and –modulator activity or sensitivity of the respective systems. (Glutamatergic and GABA-ergic systems are among the first candidates as the BAC firing, apical amplification mechanism clearly depends on the effects of these substances.).

Another line of research might be this: we can study individual differences in timing parameters of the listed phenomena as related to endophenotypes caused by genetic variability. For example, in our recent research we showed that there are individual differences in timing the magnitude of the metacontrast masking effect. Masking dynamics interacted with single nucleotid polymorphisms (SNPs) associated with serotonergic, dopaminergic and BDNF related endophenotypes (Maksimov et al., 2013, 2015a,b). This approach, if further developed, allows cellular-level precise analysis of timing of neural activity in light of behavioral effects of perceptual phenomenology. For example, the 5HTR2A mediated non-synchronous, late glutamatergic EPSP has a post-stimulation delay exceeding 50 ms. This effect is characteristic for apical dendrites of the layer-5 PNs (Marek and Aghajanian, 1998; Aghajanian and Marek, 1999a) and corresponds suitably to what Larkum (2013) has described when explaining the subcellular mechanism of cognitive binding and long range top–down modulation. Importantly, Ca^2+^ based electrogenesis of the membrane response allowing integration of the apically targeted modulatory effects and soma-directed primary sensory afference (which is central in our conceptualization) is also implicated in the slow 5-HT induced glutamatergic response (Aghajanian and Marek, 1999b). So the two approaches appear mutually consistent in describing the neuronal-level basic mechanisms of neural underpinnings of real-time cognition. Furthermore, this 5-HT based mechanism is strongly involved in hallucinogenic effects (Aghajanian and Marek, 1999a), which also suggests this mechanism is involved in visual functions. Thus, some predictions based on serotonergic system related common genetic variability could be used to reveal whether individual differences in the timing of the optima of some of the listed visual phenomena support our theoretical approach.

Visual phenomenology as related to GABA and glutamate levels in visual cortex was investigated by Terhune et al. (2015) who used transcranial magnetic stimulation for inducing phosphenes. Phosphene thresholds negatively correlated with glutamate concentrations in visual cortex (assessed by magnetic resonance spectroscopy). This result is not at odds with our theoretical stance and the views of Larkum (2013). Apical compartment activity relates to this effect.

Of course, another direction of development for the present approach could be computational modeling (e.g., Shai et al., 2014, 2015). While this remains out of the scope of the present article, I can notice some positive trends in recent published research, including the very promising approach taken by Phillips (2015). Indeed, when this article was already written to its pre-revision stage, several papers were published also suggesting that the BAC firing related apical amplification mechanism of contextual modulation could find its place at the center stage of current theorizing about conscious perception: Phillips (2015), Meyer (2015), Muckli et al. (2015). Among these, Phillips (2015) comments our earlier similar ideas (Bachmann and Hudetz, 2014) and hints at possible limitations which our account of BAC firing as the consciousness mechanism might bear. I will reply to Phillips (2015) in the part that follows.

## Unsolved Issues and Limitations of the Present Account

An obvious limitation of this paper concerns the mutual isolation of the perceptual level phenomena I explained and the mechanistic level of explanation at the (sub-)cellular level. Because of the space constraints I can not present an in-depth treatment of this issue. For a more detailed discussion of the neuronal level mechanistic description of the foundations of the present theory of the illusory phenomena different publications can be recommended (see Bachmann, 1994; Larkum, 2013; Bachmann and Hudetz, 2014). On the other hand, most of the earlier research involving cellular level description of the mechanisms featuring top–down presynaptic input and coincidence detection has been either (a) abstract computational modeling without combining direct evidence from neural/behavioral data and model behavior or (b) neurobiological experiments employing neuronal-level recording, but using anesthetized animals or *in vitro* methods. Understandably enough, this type of research can not provide data directly relevant for consciousness phenomena. Nevertheless, among the scarce research linking top–down contextually modulated perceptual behavior *in vivo* and cellular events there are studies with results consistent with the present hypothesis. Using the method of cortical cooling of V3 and V2, Nassi et al. (2013) demonstrated top–down modulation of the V1 neuronal responses in alert monkeys. Yet, the layer specificity of the lower level neurons could not be precisely ascertained with the methods of recording they used. Similarly, although the results of another monkey-study (Self et al., 2012) showed that top–down contextually driven modulations and feedforward effects are mediated differently (by NMDA receptors and AMPA receptors), the precise layer-specific description of the presynaptic afferents was not available. On the other hand, the detailed descriptions of the layered structure of the neuronal local circuits mediating perceptual and cognitive phenomena involving contextual effects have been suggested repeatedly (e.g., Grossberg and Versace, 2008). However, this research has typically followed the tradition of abstract computational modeling without directly collecting neurobiological data in association with behavioral task perfromance.

Another limitation of the present article is its “fixation” at the layer 5/6 units without explicating whether other layers of neurons may be necessary or even whether other kinds of local circuits capitalizing on the effects of neurons situated in other layers (except layer 5) could produce similar results. For example, layer 2/3 and layer 6-to-4 neurons can be assembled together so as to model a rich repertoire of the perceptual phenomena (Grossberg and Versace, 2008). Future neurobiological research combining single-cell recordings/manipulations and behavioral experiments with alert mammal subjects should help solve these dilemmas.

Phillips (2015) argues that theoretical standpoints of Bachmann and Hudetz (2014) who earlier presented the views similar to what is presented in this paper need further clarifications. First, according to Phillips (2015) it can also be argued that consciousness depends upon the formation of transient coalitions (Crick and Koch, 2003), so the role of apical amplification in creating large mutually supportive coalitions needs to be clarified. Indeed, by facilitating communication to higher cascaded levels of representation by BAC firing and therefore also creating chances of more top–down effects apical amplification also helps to invoke more pyramidal cells into forming coalitions which dissipate as soon as either somatic or apical input or both become insufficient. As Phillips (2015) rightly notices, relating assembly formation to NMDA-dependent anesthesia may be one way of doing this (Flohr et al., 1998; see also Meyer, 2015). Second, Phillips asks whether it is only the contents that are consciously experienced, but not the level, and, if so, why? I think that, directly, only contents are consciously experienced, but certain variable aspects of contents such as “clarity”, “intensity”, “fragmentariness” indirectly tell the subject what is the level of consciousness. In perceptual illusory phenomena the aspect of subjective contrast can be often noticed. Phillips (2015) also points out that strong modulatory interactions can occur even in cases where the subject is not conscious of the contextual information producing the amplification. It therefore needs to be made clear how it is possible for effective modulation to occur without consciousness. In my opinion, the level of EPSP can be shifted closer to firing threshold before firing has started and this can work selectively for certain content. This may be a kind of implicit Bayesian priors selectively modulating of what becomes selectively conscious among the possible alternatives. Actually, in most of the perceptual phenomena described in the preceding chapter this kind of subliminal influence has been assumed. Moreover, few spikes of the content-carrying layer-5 pyramidal neurons can be produced in the driving mode which may not be sufficient for consciousness, but the bursting mode caused by BAC firing is what matters. The fourth problem according to Phillips refers to our assumption that BAC firing is the mechanism by which apical input amplifies response, but the evidence reviewed in Section 2 of Phillips’ article (Phillips, 2015) suggests that other mechanisms may be more important in some classes of pyramidal cell. Of course, I think that in order to protect the apical amplification system from too long inhibited states after the preceding intensive bursting and for allowing lateral-inhibitory effects from neighboring content-specific neurons to take place, certain balancing mechanisms are needed. Phillips (2015) and Larkum (2013) list many such cellular-level mechnisms (e.g., interneurons of different types) capable of inhibition and disamplification that could optimize the work of the BAC firing integrative mechanism. On the other hand, it is possible that not all pyramidal cells that are modulated by apical amplification are part of the direct consciousness mechanisms (e,g, CA cells or some parietal cortex cells). The consciousness mechanism at its cortical level may consist in only a part of cortical pyramids such as the ones present in the higher-level occipital, most of the temporal, and some of the frontal cortex. Fifth, Bachmann and Hudetz (2014) imply that amplification can be strong enough to amplify transmission of the content, but not strong enough to elevate it into consciousness. Thus, Phillips (2015) further asks what is the difference between the apical amplification that is sufficient for consciousness and that which is not? Relying on my earlier model involving presynaptic modulation of the EPSPs of pyramidal neurons (Bachmann, 1994, 1997) and its successfully tested experimental predictions I suggest that insufficient duration of the BAC firing plateau potential may be the cause why some amplifications remain without a conscious effect.

Phillips (2015) further comments that there is a long tradition in psychiatry relating disorders such as hysteria and schizophrenia to impaired levels of consciousness and asks how is apical dendrites’ amplification related to that evidence. In my view it is possible that endophenotypes related to mental vulnerability are the result of deficient neuromediator- and –modulator systems which in turn causes abnormal phenomenology. This is what causes abnormal conscious experiences. Finally Phillips (2015) pointed out that some functions of apical amplification such as attention, working memory, and cognitive control require consciousness, but others such as priming and figure-ground segregation do not, which means that such differences at the phenomenological level require clarification. I think that both recent theoretical arguments and empirical evidence show that attention and working memory can be preconscious as well as conscious (Bachmann, 2011; Murd and Bachmann, 2011; Tsuchiya and Koch, 2016). Consequently, it seems to be a matter of degree whether and to what extent some cognitive function remains subliminal. Subliminal phase of some cognitive process can be interpreted as inter-level communication in the driving mode of neuronal spiking without sufficient generation of the plateau potentials in the bursting mode of pyramidal activity.

## Concluding Remarks

There are two big groups of neurobiological theories of the mechanisms of explicit conscious perception possibly to be used for explaining why our subjective experience of the real world is not always veridical and sometimes even deprives us from seeing distinct reality: (i) distributed global processing theories, stressing the importance of the large network and computations performed by that network (Baars, Tononi, Dehaene, Singer); (ii) local integrative modules based theories stressing the significance of the cellular and sub-cellular mechanisms for the whole global network functioning and particularly for upgrading the preconscious information processing activities in order to acquire the status of phenomenal experience of the processed contents (Llinás, Larkum, Bachmann). The group (i) does not provide specific hypotheses for the fine time scale psychophysical phenomena. Group (ii) theories are specific enough to provide experimentally testable predictions for certain psychophysical phenomena unfolding over a fine time scale. The key principle according to which the testable predictions can be formulated concerns the temporal coincidence of presynaptic inputs arriving from different cerebral regions within a critical time-window such that this satisfies the working-parameters of the BAC firing mechanism. According to this theory, the sources of presynaptic inputs to distal and basal compartments of the layer-5 neuron’s membrane that are capable of producing coincidence of these inputs are to be integrated (bound) for subjective experience. Now, the task is to see under what experimental stimulation conditions this coincidence occurs and ascertain whether the corresponding psychophysical illusory phenomenon can be thus mechanistically explained. We saw that there are indeed a couple of such phenomena. This makes the BAC firing based theory of visual phenomenology a falsifiable theory. Last but not least, expression of the subcellular and synaptic mechanisms of the brain is strongly influenced by the endophenotypes which are regulated by common genetic variability. Because this variability is associated with personality traits predictive of adaptive resilience and vulnerability, individual differences in perceptual phenomenology as based on the effects of this hypothetical mechanism might be explored with a purpose. It is – bearing in mind the possibility to develop perceptual express tests of vulnerability.

## Conflict of Interest Statement

The author declares that the research was conducted in the absence of any commercial or financial relationships that could be construed as a potential conflict of interest.
